# Enzymatic improvement of mitochondrial thiol oxidase Erv1 for oxidized glutathione fermentation by *Saccharomyces cerevisiae*

**DOI:** 10.1186/s12934-017-0658-0

**Published:** 2017-03-15

**Authors:** Jyumpei Kobayashi, Daisuke Sasaki, Kiyotaka Y. Hara, Tomohisa Hasunuma, Akihiko Kondo

**Affiliations:** 1 Graduate School of Science, Technology and Innovation, Kobe University, 1-1 Rokkodaicho, Nada-ku, Kobe, Hyogo 657-8501 Japan; 2 Graduate School of Nutritional and Environmental Sciences, University of Shizuoka, 52-1 Yada, Suruga-ku, Shizuoka, 422-8526 Japan; 3 Department of Chemical Science and Engineering, Graduate School of Engineering, Kobe University, 1-1 Rokkodaicho, Nada-ku, Kobe, Hyogo 657-8501 Japan; 4 RIKEN Center for Sustainable Resource Science, 1-7-22 Suehiro-cho, Tsurumi-ku, Yokohama, Kanagawa 230-0045 Japan

**Keywords:** Glutathione, *Saccharomyces cerevisiae*, Thiol oxidase, Mia40, Erv1

## Abstract

**Background:**

Oxidized glutathione (GSSG) is the preferred form for industrial mass production of glutathione due to its high stability compared with reduced glutathione (GSH). In our previous study, over-expression of the mitochondrial thiol oxidase *ERV1* gene was the most effective for high GSSG production in *Saccharomyces cerevisiae* cells among three types of different thiol oxidase genes.

**Results:**

We improved Erv1 enzyme activity for oxidation of GSH and revealed that S32 and N34 residues are critical for the oxidation. Five engineered Erv1 variant proteins containing S32 and/or N34 replacements exhibited 1.7- to 2.4-fold higher in vitro GSH oxidation activity than that of parental Erv1, whereas the oxidation activities of these variants for γ-glutamylcysteine were comparable. According to three-dimensional structures of Erv1 and protein stability assays, S32 and N34 residues interact with nearby residues through hydrogen bonding and greatly contribute to protein stability. These results suggest that increased flexibility by amino acid replacements around the active center decrease inhibitory effects on GSH oxidation. Over-expressions of mutant genes coding these Erv1 variants also increased GSSG and consequently total glutathione production in *S. cerevisiae* cells. Over-expression of the *ERV1* ^*S32A*^ gene was the most effective for GSSG production in *S. cerevisiae* cells among the parent and other mutant genes, and it increased GSSG production about 1.5-fold compared to that of the parental *ERV1* gene.

**Conclusions:**

This is the first study demonstrating the pivotal effects of S32 and N34 residues to high GSH oxidation activity of Erv1. Furthermore, in vivo validity of Erv1 variants containing these S32 and N34 replacements were also demonstrated. This study indicates potentials of Erv1 for high GSSG production.

**Electronic supplementary material:**

The online version of this article (doi:10.1186/s12934-017-0658-0) contains supplementary material, which is available to authorized users.

## Background

Glutathione is the most abundant thiol-containing tripeptide in all living organisms [1]. Glutathione is widely used in the medical, food, and cosmetic industries [2, 3] due to its various physiological functions such as acting as an antioxidant, a detoxifier of xenobiotics, and an immune booster [4–9]. Thus the demand for glutathione has increased in recent years. Glutathione is industrially produced mainly by fermentation using *Saccharomyces cerevisiae*, which contains a high concentration of glutathione and is accepted as a food-producing microorganism.

Glutathione is biologically synthesized by γ-glutamylcysteine (γ-GC) synthetase (GCS, EC 6.3.2.2) encoded by *GSH1* and by glutathione synthetase (GS, EC 6.3.2.3) encoded by *GSH2* from three precursor amino acids. GCS catalyzes the reaction to form γ-GC from l-glutamic acid and l-cysteine. GS catalyzes the reaction to form glutathione from γ-GC and glycine. Other glutathione-related enzymes include thiol oxidase (TO, EC 1.8.3.2) encoded by *ERV1*, *ERV2*, and *ERO1*, and glutathione-disulfide reductase (GR, EC 1.8.1.7) encoded by *GLR1*. TOs catalyze the reaction to form oxidized glutathione (GSSG) from reduced glutathione (GSH) by oxidizing the thiols, and GR catalyzes the reaction to form GSH from GSSG by reducing a disulfide bond.

In many cases, reduced glutathione (GSH) primarily exists to respond to oxidative stress in living organisms [10], and thus many glutathione-related studies have focused on GSH [11–13]. However, GSSG is of interest in glutathione production. In industrial mass production of glutathione, GSSG is preferable due to its higher stability. Furthermore, enhancements of GSSG production help to avoid a negative feedback regulation by *GSH1*, and consequently increase total glutathione production by fermentation with *S. cerevisiae* [14, 15]. GSSG also has advantages in utilization; it has the same extent of antioxidant activity in the intestines after dietary intake as GSH [16], and promotes plant growth more potently than GSH [17, 18].

In our previous study, deletion of the *GLR1* gene and over-expression of the mitochondrial thiol oxidase *ERV1* gene was the most effective for generating high GSSG production among three types of different thiol oxidase genes in *S. cerevisiae* cells [15]. Therefore, in this study, we improved the enzymatic activity of Erv1 for GSH oxidation by amino acid replacements, and consequently enhanced GSSG production in *S. cerevisiae* by using mutant *ERV1* genes.

## Methods

### Strains, media, and primers

*Saccharomyces cerevisiae* GCIΔ*GLR1*, *GSH1*/*GSH2* cocktail δ-integrated and *GLR1* deleted YPH499 (ABC1193/NBRC 10505) strain was previously constructed [19] and used for glutathione production in this study. *Saccharomyces cerevisiae* GCIΔ*GLR1* derivative strains were aerobically grown at 30 °C in yeast extract-peptonedextrose (YPD) medium (10 g l^−1^ yeast extract, 20 g l^−1^ bacto-peptone, and 20 g l^−1^ glucose) supplemented with 0.5 mg l^−1^ aureobasidin A (Aba). *Escherichia coli* NovaBlue (Novagen, Darmstadt, Germany) strain was used for DNA manipulation*. E. coli* Rosetta™(DE3)pLysS (Novagen) strain was used to produce recombinant proteins. *E. coli* strains were aerobically grown at 37 °C in Luria–Bertani (LB) medium (10 g l^−1^ tryptone, 5 g l^−1^ yeast extract, and 5 g l^−1^ sodium chloride). Ampicillin (Amp; 50 mg l^−1^) and chloramphenicol (Cm; 50 mg l^−1^) were added as necessary. The primer sequences used in this study are summarized in Additional file 1: Table S1.

### Construction of plasmids

The *ERV1* gene was amplified by polymerase chain reaction (PCR) from complementary DNA (cDNA) of *S. cerevisiae* YPH499 using primers ERV1F1 and ERV1R1. The cDNA was prepared by reverse transcription PCR using a PrimeScrip RT-PCR Kit (Takara Bio, Otsu, Japan) from total RNA extracted from *S. cerevisiae* YPH499 cells using NucleoSpin RNA (Takara Bio). The PCR product was cloned between *Sph*I and *Bam*HI sites of pUC19 (Takara Bio). After the sequence was checked, the *ERV1* gene was subcloned between *Nde*I and *Xho*I sites of pET-22b (Novagen) to give pET-*ERV1*. pET-*ERV1* was used for Erv1 protein preparation. The plasmids for preparation of Erv1 variant proteins were constructed by inverse PCR using corresponding primer pairs and templates (Additional file 1: Table S1). For expression of *ERV1* and its mutant genes in *S. cerevisiae* cells, the *ERV1* gene was amplified by PCR from pET-*ERV1* using primers ERV1F2 and ERV1R2. The PCR product was cloned between *Nhe*I and *Bam*HI sites of pGK406 designed for target gene expression in *S. cerevisiae* [20]. The mutant genes coding Erv1 variants were also amplified by the same primer pairs and cloned into pGK406.

### Plasmid introduction into *S. cerevisiae*

Derivatives of pGK406 were introduced into *S. cerevisiae* cells using the lithium acetate method [21, 22]. Transformants were selected by uracil auxotrophy. Target gene insertion into the genomic DNA of each transformant was confirmed by PCR using the appropriate primer sets.

### Preparation of recombinant proteins

The *E. coli* Rosetta™(DE3)pLysS strain harboring pET-*ERV1* was aerobically grown in 5 ml of liquid LB medium supplemented with Amp and Cm at 37 °C for 18 h. The 1 ml of grown cells was inoculated into 100 ml of liquid LB medium supplemented with Amp, Cm, and 1% lactose, and aerobically grown at 20 °C for 48 h. The grown cells were then pelleted by centrifugation (16,000×*g*, 10 min) and resuspended in 20 mM potassium phosphate buffer (pH 7.0) containing 500 mM NaCl. The cell suspension was sonicated and centrifuged (16,000×*g*, 10 min) to remove cell debris. The His-tagged Erv1 protein in the supernatant was purified by TALON^®^ Metal Affinity Resin (Takara Bio). The Erv1 variants were also prepared by the same method.

### Enzyme assay

The activities of recombinant Erv1 and its variants were determined by measuring the initial velocity of product formation. The assay mixture containing 50 mM potassium phosphate buffer (pH 7.0), 10 mM GSH or γ-GC, and the purified recombinant protein was incubated at 30 °C for 10 min. The reaction was then stopped by adding an equal volume of 20% (w/v) trichloroacetic acid. The formed products were measured using high performance liquid chromatography (HPLC) as mentioned below. The protein concentrations were assayed by the Bradford method. All assays were separately performed three times.

### Glutathione production

The *S. cerevisiae* GCIΔ*GLR1*/*ERV1* strain was streaked and grown on YPD solid media with Aba at 30 °C for 72 h. A single grown colony was inoculated into 5 ml of YPD liquid medium with Aba and aerobically grown at 30 °C for 18 h. The grown cells were inoculated into 20 ml of the same medium. The initial cell density (OD_600_) was 0.03, and cells were grown in a 200 ml baffled Erlenmeyer flask at 30 °C with agitation at 150 rpm for up to 48 h. The other mutant strains were grown by the same method. Intracellular GSH and GSSG were analyzed by HPLC as mentioned below. All fermentations for glutathione production were separately performed three times.

### Analytical methods

To determine the cell concentration, the OD_600_ of the culture sample was measured using an UVmini-1240 Spectrometer (Shimadzu, Kyoto, Japan). Samples were prepared according to a previous report [19]. GSH and GSSG concentrations were determined by HPLC (Shimadzu) equipped with the YMC-Pack ODS-A column (YMC, Kyoto, Japan). The operating condition was 30 °C, with 50 mM potassium dihydrogen phosphate buffer (pH 2.8) and 10 mM sodium 1-heptanesulfonate as the mobile phase at a flow rate of 1.0 ml min^−1^, and detection was performed with a UV detector SPD-20A (Shimadzu) at 210 nm. Intracellular (reduced, oxidized, and total) glutathione content was calculated using the volumetric glutathione concentration (g l^−1^) divided by cell concentration (OD_600_ × 0.3) (g l^−1^), represented as % values (w/w).

### Protein modeling

Protein structures and intramolecular interactions were simulated using PyMol software (https://www.pymol.org/). The structural data of the Erv1 variant protein of *S. cerevisiae* (PDB ID: 4E0I) was retrieved from RCSB Protein Data Bank (http://www.rcsb.org/pdb/home/home.do) and used for constructions of Erv1 and its variants structures.

## Results

### Catalytic activity of Erv1 and its variants

**Fig. 1:**
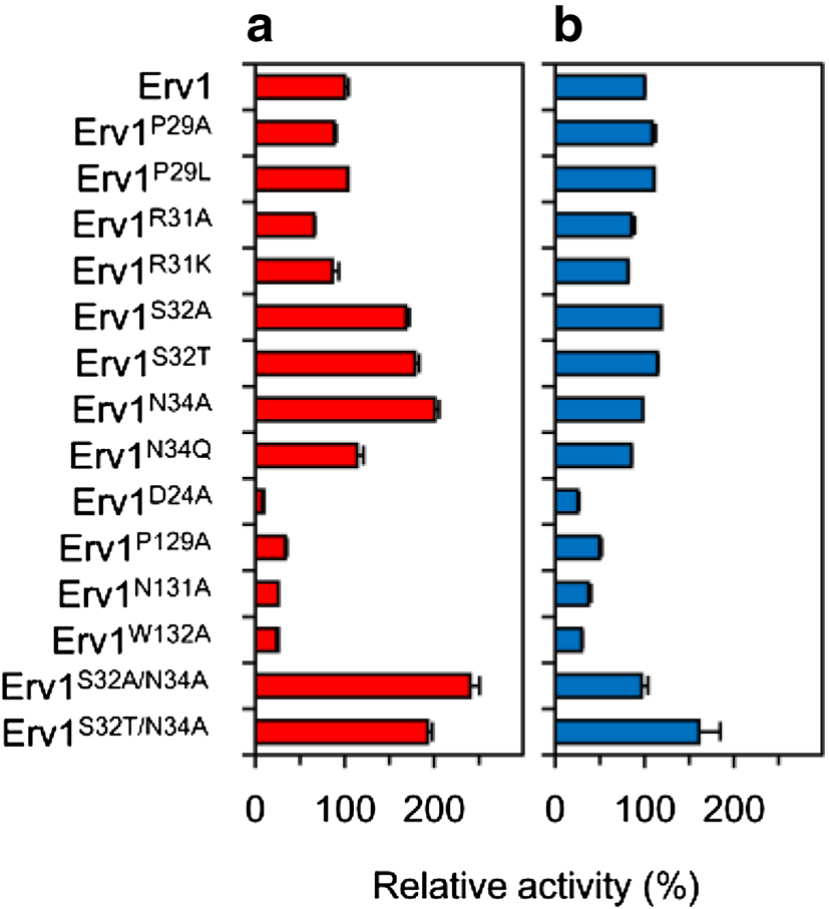
Relative activities of Erv1 and its variants. **a** GSH was used as a substrate; **b** γ-GC was used as a substrate. Erv1 and variants were assayed for 10 min at 30 °C and pH 7.0 (potassium phosphate buffer) using 10 mM substrates. The 100% relative activities of Erv1 using GSH and γ-GC as substrates were 1.62 and 1.59 mU mg^−1^, respectively. All assays were separately performed three times. Data are presented as the mean ± standard deviation (n = 3)

**Fig. 2:**
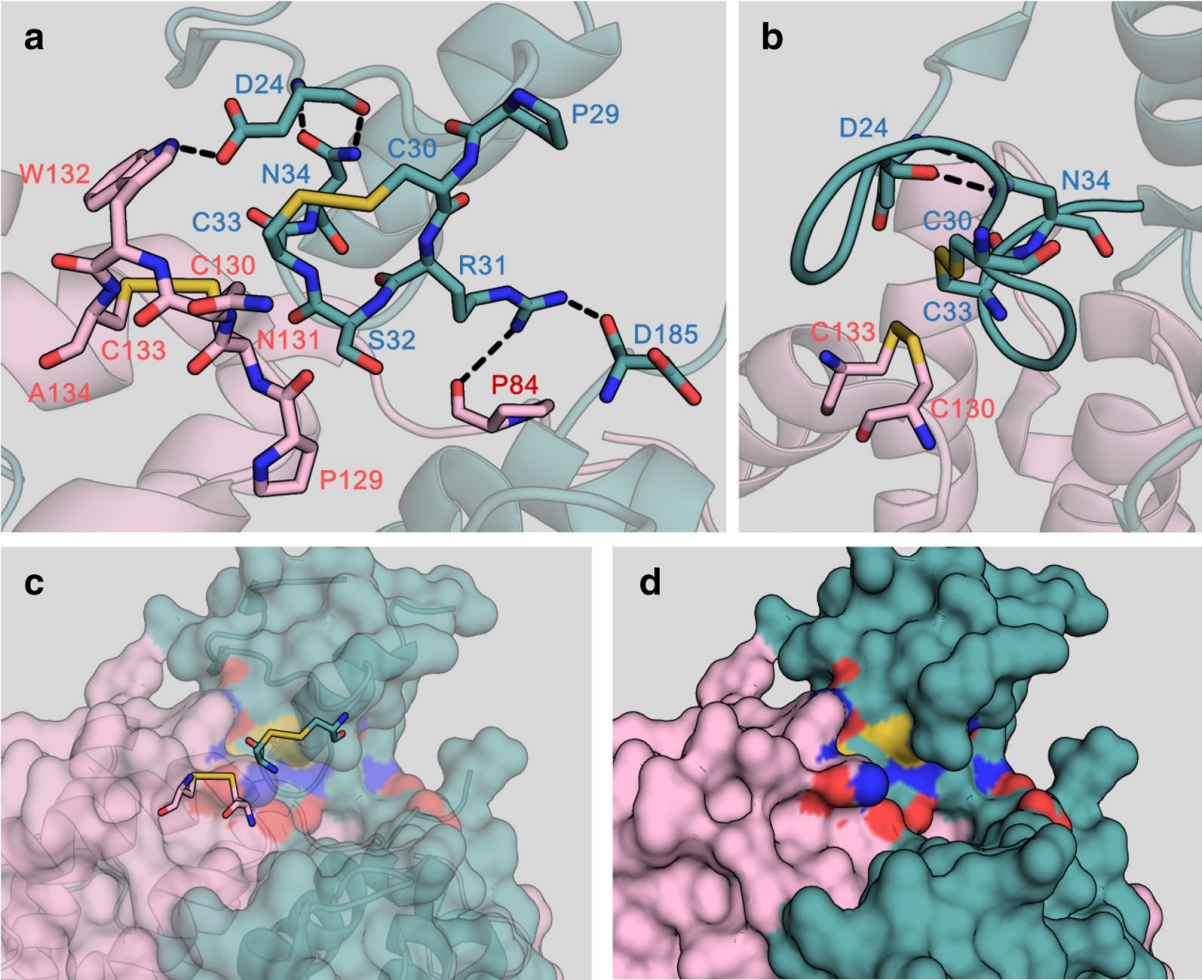
Structural models of the area surrounding catalytic cysteine residues.  **a** Catalytic cysteine and its surrounding residues; **b** lateral view of the catalytic site; **c**, **d** surface around the active site. The structures were constructed from Erv1 variant protein of *S. cerevisiae* (PDB ID: 4E0I) by PyMOL software. Each subunit was separately rendered in *green* and *pink*. Oxygen, nitrogen, and sulfur atoms are rendered in *red*, *blue*, and *yellow*, respectively. Hydrogen bonds are shown as *black dashed lines*

*ERV1* and its mutants were successfully expressed in *E. coli* cells. The gene products were subsequently purified to homogeneity by metal affinity column chromatography. To evaluate their catalytic profiles, we first prepared and assayed Erv1 and its variants containing a replaced residue next to the catalytic cysteines (C30 and C33). Erv1^S32A^, Erv1^S32T^, and Erv1^N34A^ oxidized GSH more efficiently than Erv1 (relative activity, 169, 178, and 201%, respectively) (Fig. 1a), whereas they oxidized γ-GC at comparable rates (relative activity, 119, 114, and 98%, respectively) (Fig. 1b). The other variants oxidized GSH and γ-GC at comparable or decreased rates (Fig. 1a, b). From the Erv1 protein structure, the side chain of N34 forms two hydrogen bonds to the main chain of D24, and the side chain of D24 also forms a hydrogen bond to the side chain of W132 located between C130 and C133, which are other catalytic cysteine residues (Fig. 2a, b) [23]. This fact suggests that these hydrogen bonds and the surrounding residues C130 and C133 play pivotal roles in the enzymatic oxidation of GSH. To investigate these implications, we additionally prepared and assayed Erv1^D24A^, Erv1^P129A^, Erv1^N131A^, and Erv1^W132A^, and these variants oxidized GSH and γ-GC at much lower rates (Fig. 1a, b) than did Erv1. Therefore, S32A, S32T, and N34A mutations are valuable for increasing Erv1 activity for GSH.

To obtain effective Erv1 variants, we further prepared Erv1^S32A/N34A^ and Erv1^S32T/N34A^, and assayed their specific activities. Erv1^S32A/N34A^ showed the highest relative activity for GSH (240%) among all variants, and showed comparable activity for γ-GC (96%). On the other hand, Erv1^S32T/N34A^ showed almost the same activity for GSH (192%) compared to Erv1^S32A^, Erv1^S32T^, and Erv1^N34A^, and high activity for γ-GC (161%).

### Thermal and kinetic profiles of Erv1 and its variants

**Fig. 3:**
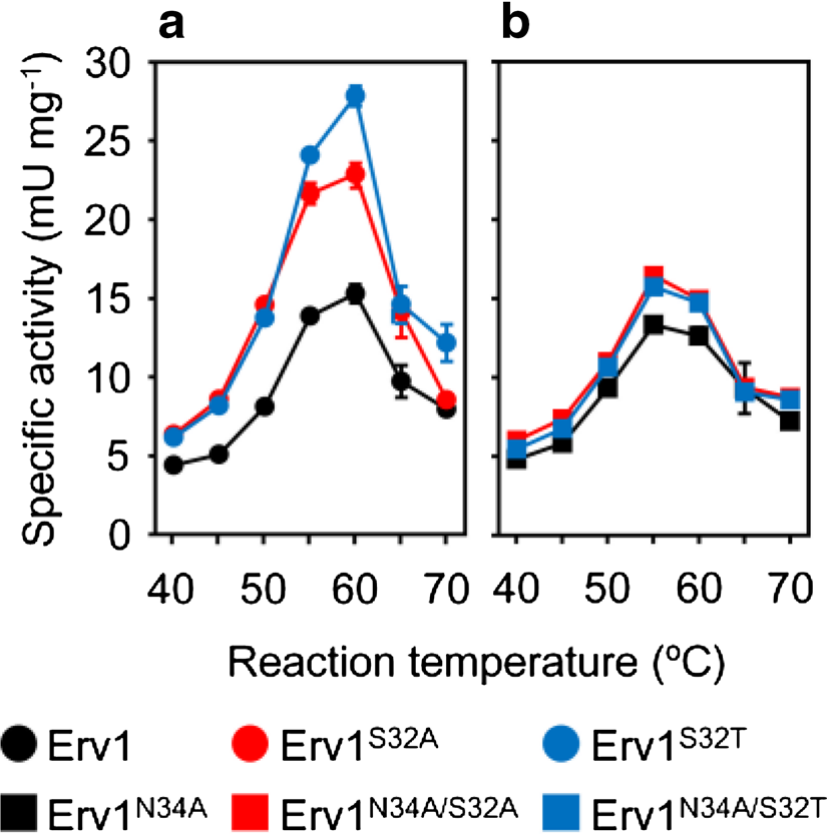
Temperature effect on enzyme activity. **a** Specific activities of Erv1, Erv1^S32A^ and Erv1^S32T^; **b** specific activities of Erv1^N34A^, Erv1^S32A/N34A^ and Erv1^S32T/N34A^. Data are presented as the mean ± standard deviation (n = 3)

To investigate the effect of mutations on the Erv1 structure, thermal profiles of Erv1 and its variants that showed higher activities for GSH than parent Erv1 were assessed. Erv1, Erv1^S32A^, and Erv1^S32T^ exhibited maximum activities at 60 °C (15.3, 22.9, and 27.8 mU mg^−1^, respectively) (Fig. 3a; Table 1). Erv1^S32A^ and Erv1^S32T^ were more activated by high temperature than parental Erv1. On the other hand, Erv1^N34A^, Erv1^S32A/N34A^, and Erv1^S32T/N34A^ exhibited similar profiles to that of parental Erv1 with slightly decreased maximum temperatures at 55 °C (13.3, 16.5, and 15.8 mU mg^−1^, respectively) (Fig. 3b; Table 1). Thermostabilities of Erv1 and its variants were also assessed by incubating enzyme solutions for 1 h at each temperature before assaying. All tested variants showed obvious decreases in thermostability, whereas parental Erv1 was much more robust (Table 1). The variants containing N34A replacement showed lower thermostabilities than those of the variants containing S32 replacements (Table 1). These results suggest that both S32 and N34 residues greatly contribute not only to oxidations of GSH but also to thermostability of the enzyme.

**Table 1:** Kinetic and thermal profiles of Erv1 and its variants

Protein	Maximum temperature (°C)^a^	*T*_1/2_ (°C)^b^	*K*_*m*_ (mM)^c^	*V*_*max*_ (mU)^c^	*K*_*cat*_ (min^−1^)^c^
Erv1	60	<90	4.4	2.1	0.05
Erv1^S32A^	60	69.3	13.0	5.4	0.12
Erv1^S32T^	60	68.8	12.0	4.4	0.10
Erv1^N34A^	55	64.1	33.5	11.6	0.25
Erv1^S32A/N34A^	55	59.1	40.5	15.9	0.42
Erv1^S32T/N34A^	55	62.0	41.2	9.1	0.20

^a^The enzymes were assayed at each temperature and pH 7.0 for 10 min with 10 mM GSH as the substrate

^b^The temperature at which the residual activity equaled 50%. The enzymes were incubated at each temperature and subsequently assayed at 30 °C and pH 7.0 for 10 min with 10 mM GSH as the substrate

^c^The enzymes were assayed at 30 °C and pH 7.0 for 10 min with various concentrations of GSH as the substrate

The kinetic profiles of Erv1 and its variants were also analyzed. All variants exhibited higher *K*_*m*_, *V*_*max*_, and *K*_*cat*_ values than the parental enzyme (Table 1). The Erv1^S32A^ and Erv1^S32T^ containing only S32 replacement showed about 2.7- to 3.0- and 2.1- to 2.6-fold higher *K*_*m*_ and *V*_*max*_ values than those of the parental Erv1, respectively (Table 1). Furthermore, Erv1^N34A^, Erv1^S32A/N34A^ and Erv1^S32T/N34A^ containing N34A replacement showed about 7.6- to 9.4- and 4.3- to 7.6-fold higher *K*_*m*_ and *V*_*max*_ values than those of the parental enzyme, respectively (Table 1). These differences in thermal and kinetic profiles between variants containing S32 and/or N34 replacements suggest that effect of the N34A replacement to the Erv1 profile was greater than the S32 replacements.

### Glutathione production by recombinant *S. cerevisiae* strains

**Fig. 4:**
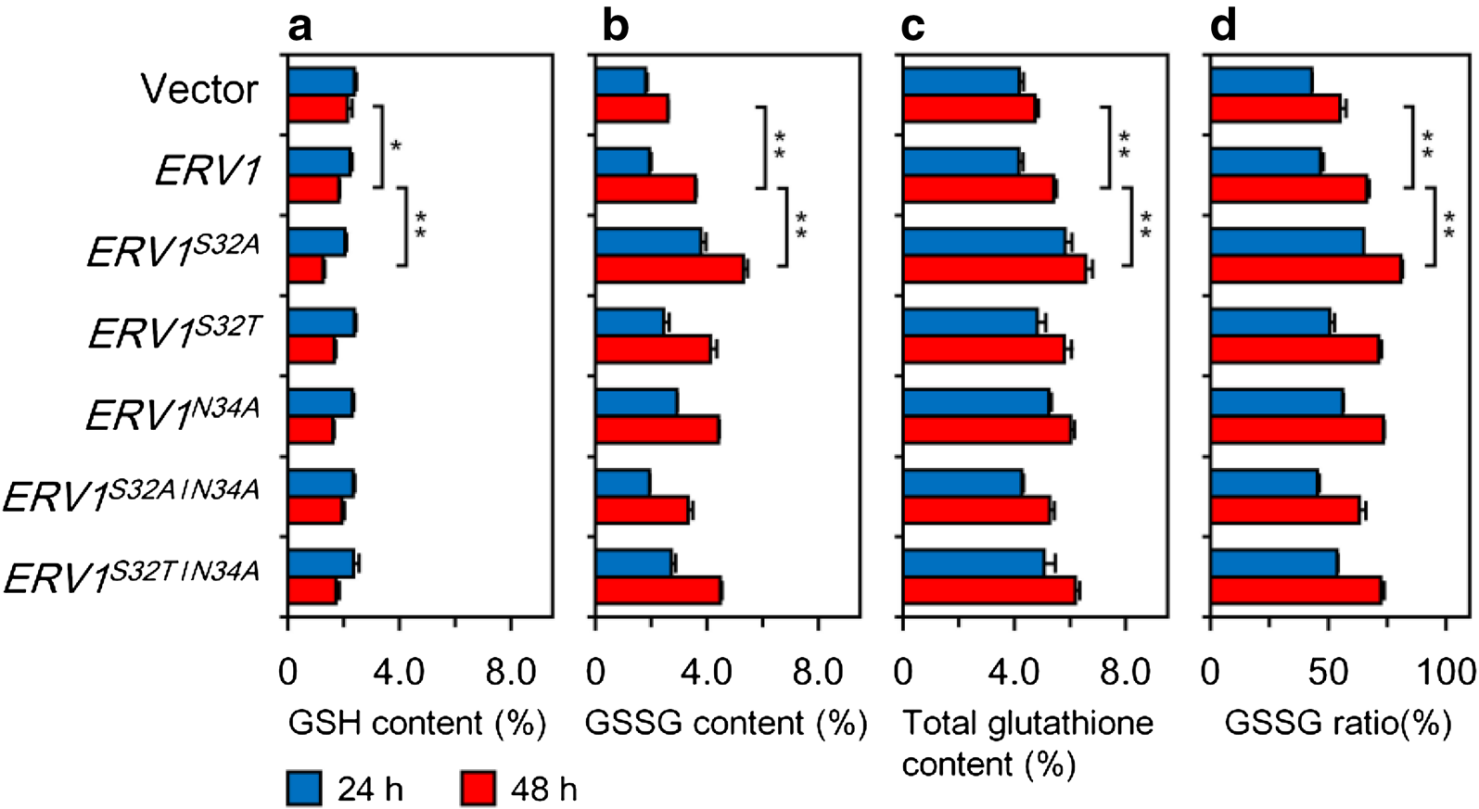
Glutathione production by *S. cerevisiae* GCIΔ*GLR1* strains over-expressing *ERV1* and its mutant genes. **a** Intracellular GSH content; **b** intracellular GSSG content; **c** intracellular GSH and GSSG content; **d** intracellular GSSG ratio to GSH and GSSG. All fermentation for glutathione production were separately performed three times. Data are presented as the mean ± standard deviation (n = 3). *Asterisks* indicate statistical significances determined by Student’s *t* test. *One asterisk* indicates a p value smaller than 0.05 (p < 0.05). *Two asterisks* indicate a p value smaller than 0.01 (p < 0.01)

To investigate the effects of mutant *ERV1* genes for glutathione production by the *S. cerevisiae* GCIΔ*GLR1* strain, derivative strains expressing *ERV1* and its mutant genes were constructed and grown. When the strains were grown for 24 h, GCIΔ*GLR1*/*ERV1* ^*S32A*^ produced much more intracellular GSSG (3.79%) and total glutathione (5.82%) than those of the other strains (Fig. 4b, c), whereas other derivative strains, especially GCIΔ*GLR1*/*ERV1*, produced comparable GSH, GSSG and total glutathione to those of the vector control strain (Fig. 4). On the other hand, most of all strains expressing mutant *ERV1* genes produced more intracellular GSSG and total glutathione than those of GCIΔ*GLR1*/*ERV1* at 48 h (Fig. 4b, c). Unlike the results of in vitro enzyme assays, GCIΔ*GLR1*/*ERV1* ^*S32A*^ produced the highest intracellular GSSG (5.30%) and total glutathione (6.57%) at 48 h (Fig. 4b, c). GCIΔ*GLR1*/*ERV1* ^*S32T*^, GCIΔ*GLR1*/*ERV1* ^*N34A*^, and GCIΔ*GLR1*/*ERV1* ^*S32T/N34A*^ produced only modestly high intracellular GSSG (4.13, 4.41, and 4.48%, respectively) and total glutathione (5.78, 6.02, and 6.20%, respectively) compared to GCIΔ*GLR1*/*ERV1* ^*S32A*^ (Fig. 4b, c). GCIΔ*GLR1*/*ERV1* ^*S32A/N34A*^ produced lower GSSG (3.32%) and total glutathione (5.26%) compared to GCIΔ*GLR1*/*ERV1*, whereas the in vitro activity of its gene product for GSH was the highest among parental and other variant proteins (Figs. 1a, 4b, c). These variant strains also showed high volumetric GSSG and total glutathione production, though they showed slightly poor growth compared to the parental strain (Table 2). GCIΔ*GLR1*/*ERV1* ^*S32T/N34A*^ produced comparable volumetric total glutathione (105.7 mg l^−1^) to that of GCIΔ*GLR1*/*ERV1* ^*S32A*^ (105.5 mg l^−1^) due to their higher cell growth (1.71 g-cell l^−1^) than that of GCIΔ*GLR1*/*ERV1* ^*S32A*^ (1.61 g-cell l^−1^) (Table 2). However, GCIΔ*GLR1*/*ERV1* ^*S32A*^ also showed the highest volumetric GSSG (85.2 mg l^−1^) production among all tested strains (Table 2).

**Table 2:** Growth and volumetric glutathione production of *ERV1*- and mutant-expressing strains

Expressing gene	Cell growth (g-cell l^−1^)	GSH (mg l^−1^)^a^	GSSG (mg l^−1^)^a^	Total glutathione (mg l^−1^)^a^
Vector	1.87 ± 0.07 (100)	38.7 ± 4.0 (100)	47.1 ± 1.0 (100)	85.8 ± 4.7 (100)
*ERV1*	1.77 ± 0.03 (95)	32.3 ± 0.6 (83)	63.4 ± 2.7 (135)**	95.8 ± 3.3 (112)*
*ERV1* ^*S32A*^	1.61 ± 0.04 (86)*	20.3 ± 1.1 (52)**	85.2 ± 2.7 (181)**	105.5 ± 5.9 (123)**
*ERV1* ^*S32T*^	1.70 ± 0.11 (91)	28.3 ± 2.6 (73)*	70.5 ± 8.4 (150)**	98.8 ± 10.9 (115)
*ERV1* ^*N34A*^	1.64 ± 0.14 (88)	26.3 ± 2.8 (68)*	72.3 ± 7.1 (154)**	98.7 ± 9.9 (115)
*ERV1* ^*S32A/N34A*^	1.83 ± 0.13 (98)	35.5 ± 3.8 (92)	60.6 ± 1.3 (129)**	96.2 ± 4.5 (112)*
*ERV1* ^*S32T/N34A*^	1.71 ± 0.05 (91)*	29.3 ± 2.6 (76)*	76.4 ± 2.9 (162)**	105.7 ± 5.2 (123)**

Culture times are 48 h. Culture conditions are described in the "Methods" section. All fermentations for glutathione production were separately performed three times. All values are represented as mean ± standard deviation (n = 3). Parentheses represent the relative values (%)

Asterisks indicate statistical significances determined by Student’s t test. * p < 0.05. ** p < 0.01

^a^Volumetric GSH, GSSG, and total glutathione (GSH and GSSG) concentrations were calculated from the cell concentrations and each cellular content as shown in Fig. 4a–c

## Discussion

**Fig. 5:**
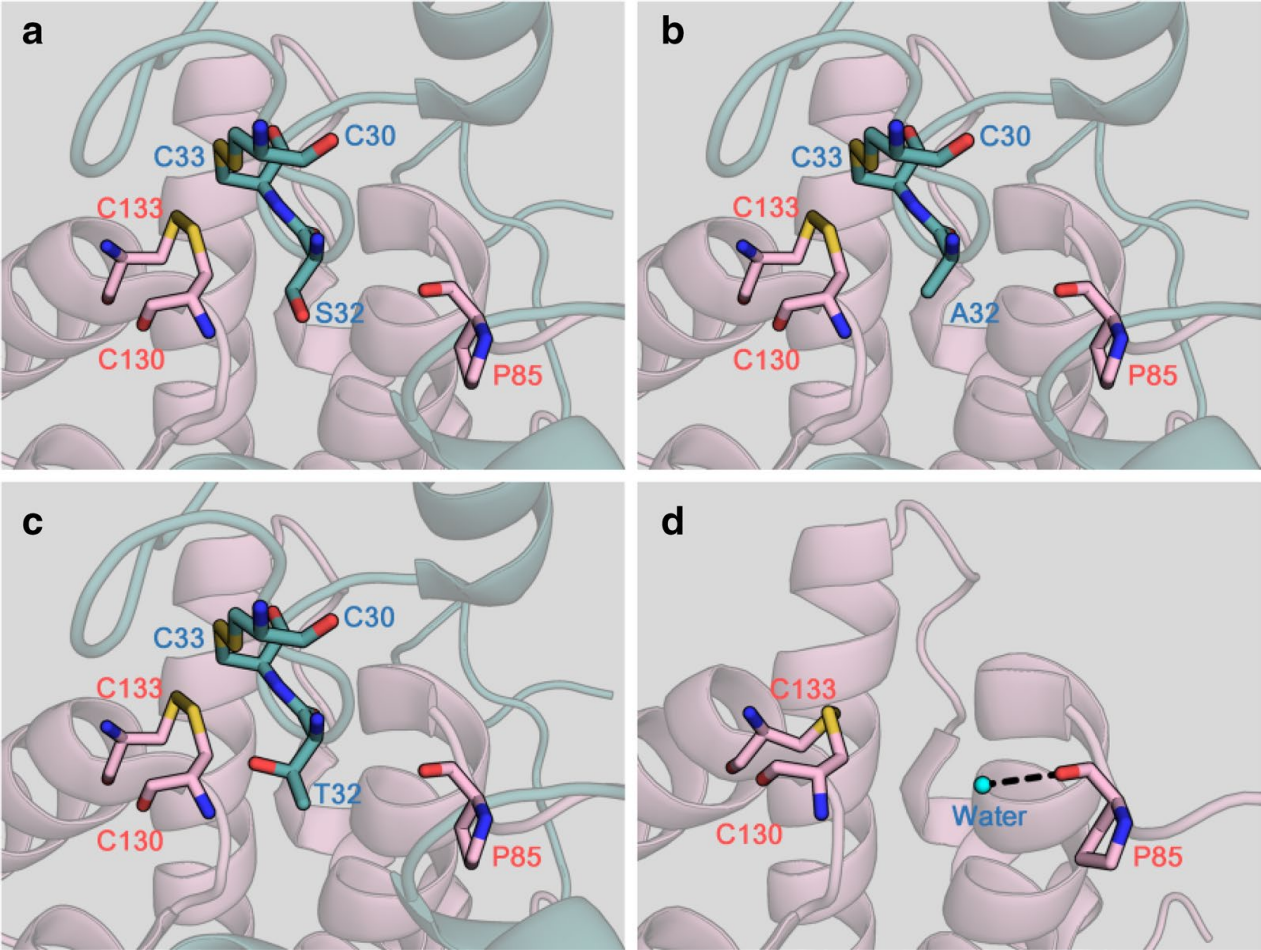
Lateral view of the area surrounding catalytic cysteine residues of Erv1 and its variants. **a** Erv1; **b** Erv1^S32A^; **c** Erv1^S32T^; **d** different rendering of Erv1 with solvents. The structures were constructed from Erv1 mutant protein of *S. cerevisiae* (PDB ID: 4E0I) (**a**–**c**) and the Erv1 core of *S. cerevisiae* (PDB ID: 3W4Y) (**d**) by PyMOL software. Each subunit was separately rendered in *green* and *pink*. Oxygen, nitrogen, and sulfur atoms are rendered in *red*, *blue*, and *yellow*, respectively. The hydrogen bond and water molecule are shown as a *black dashed line* and cyan sphere, respectively

The *ERV1* gene of *S. cerevisiae* has been studied mainly for its physiological roles due to its essentiality for respiration and growth [24–26]. In a series of studies, the active residues, cofactor binding residues, and three-dimensional structures of Erv1 have been revealed [23, 27, 28]. In our previous study, we first focused on the *ERV1* gene for biomaterial production, and revealed that over-expression of the *ERV1* gene enhanced GSSG and total glutathione production in *S. cerevisiae* cells [15]. In this study, we also first improved Erv1 for biomaterial production and revealed that S32 and N34 residues are critical for oxidation of GSH and protein stability. The side chain of N34 forms two hydrogen bonds to the main chain of D24 located in the loop covering the catalytic cysteines (Fig. 2a, b). The deletion of these hydrogen bonds by the N34A replacement led to an enhancement of Erv1 catalytic activity for GSH (Fig. 1a), whereas the catalytic activity for γ-GC remained unchanged. This result suggests that this covering loop inhibits oxidation of GSH. This idea is also supported by the comparable relative activities of Erv1^N34Q^ for GSH (114%), because the side chain of Q34 in PyMol simulations also forms hydrogen bonds to the main chain of D24, similar to the hydrogen bonds between N34 and D24 in parental Erv1 (data not shown). The comparable activities of Erv1^N34A^ and Erv1^N34Q^ for γ-GC (98 and 85%, respectively) also support this idea. Inhibition by the covering loop for γ-GC may be lower than for GSH because γ-GC, a precursor of GSH, is a smaller molecule and has similar properties. On the other hand, Erv1^S32A^ and Erv1^S32T^ also showed high oxidation activities for GSH (Fig. 1a), though no interaction was found between S32 and other residues. In addition, the thermostabilities of these variants were obviously lower than that of parental Erv1 (Table 1). These results imply that the S32 residue interacts with other residues through solvent or metal ions. Therefore, we searched for and found an incomplete three-dimensional structure of *S. cerevisiae* Erv1 with solvents (Protein ID: 3W4Y). In this structure, a water molecule is located between corresponding S32 and P85 residues, and the main chain of P85 forms a hydrogen bond to the water molecule (Fig. 5d). This implies that the side chain of S32 and main chain of P85 form a hydrogen bond through a water molecule. However, Erv1^S32T^ also showed similar activity and thermostability profiles to Erv1^S32A^, though the side chain of T32 in Erv1^S32T^ also contains a hydroxyl group like the S32 residue in parental Erv1. In PyMol simulations, steric hindrance when the methyl moeity of the side chain of T32 was facing outward from the active center was small due to its bulkiness (Fig. 5c). This suggest that the hydrogen bond through a water molecule between S32 and P85 residues in Erv1 was deleted by the S32A and S32T replacements, and that these deletions resulted in increased flexibility around the active center and reduced steric hindrance for GSH (Fig. 2c, d).

The kinetic profiles of Erv1 and its variants provided interesting insights into mechanisms of GSH oxidation by Erv1 variants (Table 1). Generally, increasing of *K*_*m*_ value indicates decreasing of substrate binding affinities. However, *V*_*max*_ values of Erv1 variants were also increased, and the over-expressions of mutant genes coding these Erv1 variants practically increased GSSG production in *S. cerevisiae* cells. These facts imply that Erv1 variants became hard to be binding to GSH compared with parental Erv1, due to instabilizations around the catalytic center by amino acid replacements. However, rates of GSH oxidations and/or GSSG dissociations from Erv1 variants became fast by decreasing of inhibitory effects as mentioned above.

The over-expression of mutant *ERV1* genes also increased GSSG and total glutathione production in *S. cerevisiae* cells as expected (Fig. 4b, c). These strains also showed higher volumetric GSSG and total glutathione production, whereas decreases in growth were observed in these recombinant strains (Table 2). In Table 2, the relationship between cell growth and GSH production is seemed to be proportional. This relationship is probably caused by reduced redox potentials of GSH in engineered strains. Generally, GSH works as a redox and antidotal agent in cells and therefore is an essential for various biological activities in all organisms [1]. Indeed, *GSH1* or *GSH2* deleted mutant *S. cerevisiae* strains lost their growth abilities in growth media without GSH [29], and genetically engineered *S. cerevisiae* strains that produce high concentration of GSH exhibited significant tolerance against diverse stresses such as high temperatures and presence of toxic agents [30, 31].

The in vivo GSSG production of the recombinant strains expressing *ERV1* and its mutant genes was not necessarily reflected by in vitro enzyme activities (Figs. 1a, 4b). Generally, thiol oxidase oxidizes various substrates that are not only small molecules [27, 28], but also macromolecules such as mitochondrial intermembrane space (MIMS) proteins including Mia40, Cox19, and so on [24, 25]. Therefore, the Erv1 variants, especially Erv1^S32A/N34A^, may oxidize unexpected substrates and form disulfide bonds between GSH and thiols in other substrates in *S. cerevisiae* cells, due to a change in substrate specificity by the amino acid replacements. Indeed, the existence of unproductive oxidized MIMS proteins [25] and a wide variety of protein-glutathione adducts in yeast cells [32] have been reported in previous studies. Generally, Mia40 oxidizes thiols in proteins, and is consequently oxidized by Erv1 in the MIMS [26]. In this study, Erv1 protein was successfully improved for oxidation of GSH, and over-expression of its coding genes meaningfully increased GSSG production in *S. cerevisiae* cells. These results suggest that the Erv1 protein directly oxidized GSH in *S. cerevisiae* cells. The suppression of GSSG production by the simultaneous over-expression of *ERV1* and *MIA40* genes in our previous study [15] and the much lower oxidation activity of Mia40 for GSH [25] also support this idea.

GSSG and total glutathione production of *S. cerevisiae* were increased by over-expressing improved *ERV1* genes. However, glutathione has been produced by industrial fermentation methods using high glutathione producing strains such as *Candida utilis* and *S. cerevisiae*, and their glutathione productions have been improved by screening from randomly mutated strains and metabolic engineering. These improved strains produce much higher amount of glutathione (e.g., *S. cerevisiae* K-2 strain produces 2700 mg l^−1^ glutathione for 24 h) than the host strain used in this study [3]. However, in many cases for improving glutathione production by metabolic engineering, genes involved in synthesis of GSH and its precursors, such as *GSH1*, *PRO1*, and *CYS3* are frequently focused [19, 30, 33]. On contrast, *ERV1* has never been applied for industrial glutathione production, and therefore Erv1 has even greater potential for industrial GSSG production when *ERV1* and its mutant genes were overexpressed in the industrial glutathione producing *S. cerevisiae* host strain.

## Conclusions

In this study, mitochondrial thiol oxidase Erv1 was applied for improvement of GSSG production by *S. cerevisiae*, and its enzyme activity for GSH oxidation was improved for the first time by site-directed mutations. The critical roles of S32 and N34 residues for GSH oxidation and protein stability were revealed with possible reasons. Five engineered Erv1 variants containing S32 and/or N34 replacements exhibited about 1.7- to 2.4-fold higher in vitro GSH oxidation activity than that of the parental Erv1. The over-expression of mutant *ERV1* genes coding these variants also demonstrated in vivo validity, showing 1.5-fold higher GSSG production than that of the strain over-expressing *ERV1* gene. This study indicates potential of Erv1 for high GSSG production by *S. cerevisiae*.

## Additional file

**Additional file 1: Table S1.** Primer sequences, restriction sites, and DNA templates used for plasmid constructions in this study are summarized.

## Abbreviations

Aba A: aureobasidin A
Amp: ampicillin
cDNA: complementary deoxyribonucleic acid
Cm: chloramphenicol
DNA: deoxyribonucleic acid
GCS: γ-glutamylcysteine synthetase
GR: glutathione-disulfide reductase
GS: glutathione synthetase
GSH: reduced glutathione
GSSG: oxidized glutathione
HPLC: high performance liquid chromatography
LB: Luria–Bertani
MIMS: mitochondrial intermembrane space
PCR: polymerase chain reaction
RNA: ribonucleic acid
TO: thiol oxidase
YPD: yeast extract-peptonedextrose
γ-GC: γ-glutamylcysteine
